# Effects of a Carob-Pod-Derived Sweetener on Glucose Metabolism

**DOI:** 10.3390/nu10030271

**Published:** 2018-02-27

**Authors:** Carmen Lambert, Judit Cubedo, Teresa Padró, Gemma Vilahur, Sergi López-Bernal, Milagros Rocha, Antonio Hernández-Mijares, Lina Badimon

**Affiliations:** 1 Program ICCC-Cardiovascular Research Center, Institut de Reserca, Hospital de la Santa Creu i Sant Pau, IIB-Sant Pau, 08025 Barcelona, Spain; clambert@santpau.cat (C.L.); jcubedo@santpau.cat (J.C.); tpadro@santpau.cat (T.P.); gvilahur@santpau.cat (G.V.); slopezb@santpau.cat (S.L.-B.); 2 Centro de Investigación Biomédica en Red de Enfermedades Cardiovasculares, Ciber CV, Instituto de Salud Carlos III, 28029 Madrid, Spain; 3 Service of Endocrinology, University Hospital Dr Peset, Foundation for the Promotion of Health and Biomedical Research in the Valencian Region (FISABIO), 46020 Valencia, Spain; Milagros.Rocha@uv.es (M.R.); hernandez_antmij@gva.es (A.H.-M.); 4 Department of Medicine, University of Valencia, 46010 Valencia, Spain; 5 Cardiovascular Research Chair, UAB, 08025 Barcelona, Spain

**Keywords:** impaired glucose tolerance, type 2 diabetes mellitus, sweetener, insulin-like growth factor, C4A complement

## Abstract

Background: Patients with type 2 diabetes mellitus (T2DM) have a higher incidence of cardiovascular (CV) events. The ingestion of high-glycemic index (GI) diets, specially sweetened beverage consumption, has been associated with the development of T2DM and CV disease. Objective: We investigated the effects of the intake of a sweetened beverage, obtained from natural carbohydrates containing pinitol (PEB) compared to a sucrose-enriched beverage (SEB) in the context of impaired glucose tolerance (IGT) and diabetes. Methods: The study was divided in three different phases: (1) a discovery phase where the plasma proteomic profile was investigated by 2-DE (two-dimensional electrophoresis) followed by mass spectrometry (matrix-assisted laser desorption/ionization time-of-flight—MALDI-TOF/TOF) in healthy and IGT volunteers; (2) a verification phase where the potential mechanisms behind the observed protein changes were investigated in the discovery cohort and in an additional group of T2DM volunteers; and (3) the results were validated in a proof-of-concept interventional study in an animal model of diabetic rats with complementary methodologies. Results: Six weeks of pinitol-enriched beverage (PEB) intake induced a significant increase in two proteins involved in the insulin secretion pathway, insulin-like growth factor acid labile subunit (IGF1BP-ALS; 1.3-fold increase; *P* = 0.200) and complement C4A (1.83-fold increase; *P* = 0.007) in IGT subjects but not in healthy volunteers. Changes in C4A were also found in the serum samples of Zucker diabetic fatty (ZDF) rats after four weeks of PEB intake compared to basal levels (*P* = 0.042). In addition, an increased expression of the glucose transporter-2 (GLUT2) gene was observed in the jejunum (*P* = 0.003) of inositol-supplemented rats when compared to sucrose supplementation. This change was correlated with the observed change in C4A (*P* = 0.002). Conclusions: Our results suggest that the substitution of a common sugar source, such as sucrose, by a naturally-based, pinitol-enriched beverage induces changes in the insulin secretion pathway that could help to reduce blood glucose levels by protecting β-cells and by stimulating the insulin secretion pathway. This mechanism of action could have a relevant role in the prevention of insulin resistance and diabetes progression.

## 1. Introdution

Diabetes is a chronic degenerative disease [1], characterized by hyperglycaemia caused by defects in insulin activity, secretion, and/or metabolism [2], that has become an epidemic and is now one of the biggest health challenges [3]. Type 2 diabetes mellitus (T2DM) represents the vast majority of cases worldwide [2] and millions of people are at high risk of developing T2DM, or suffering impaired glucose tolerance (IGT), which is a prediabetes state [4]. The relationship between T2DM and cardiovascular disease (CVD) is well established, since patients with diabetes mellitus have an increased risk of developing CVD [5,6]. Recent prospective studies have linked a food high-glucose index (GI) diet in the pathogenesis of CVD, T2DM, and obesity [7,8], and carbohydrate intake with an elevated risk of mortality [9]. Several studies have shown a link between the consumption of beverages with artificial and/or natural sweeteners and the risk of developing T2DM [10], related metabolic disorders [11] and CVD [12,13]. Beverages with carbohydrate-based sweeteners promote weight gain and contribute to an increased risk of T2DM not only through obesity but also due to the glycaemic effects derived from the consumption of large amounts of rapidly absorbable sugars. Interest has focused on recommending the intake of low-GI beverages with natural sweeteners to both prevent the development of diabetes and effectively manage diabetic patients [14,15]. A continuous association between fasting blood glucose levels and CVD, in both diabetic and non-diabetic patients, has suggested that all individuals may benefit from blood glucose level lowering strategies.

A new carbohydrate-based beverage extracted from carob pods containing pinitol, has been shown to improve the postprandial glycaemic response in healthy individuals as opposed to sucrose-based beverages [16,17]. Normal western diets provide between 0.5 g and 0.7 g of inositol per day, mainly through vegetables and fruits [18]. Reduced pinitol levels were observed in impaired glucose tolerance and diabetic patients [19]. To prevent or ameliorate diabetes mellitus (DM), the normal supplementation dose of inositol is between 2 g/day and 4 g/day [20]. The aim of the present study was to identify by proteomic approaches and experimental proof-of-concept studies, the potential mechanisms behind improvement in the glycaemia response pattern associated with the regular and chronic ingestion of a pinitol-enriched beverage.

## 2. Materials and Methods

### 2.1. Human Study

#### 2.1.1. Subjects and Study Design

Two groups of volunteers were studied. Healthy volunteers (HV; *N* = 40) and overweight volunteers with impaired glucose tolerance (IGT; *N* = 40). A third group of T2DM (*N* = 38) patients was also included in the validation phase. All subjects were recruited at the University Hospital Dr. Peset, Valencia and the study was designed as previously described [16,17,21,22].

Briefly, the subjects included in the study had an age range of 18–72 years; a body mass index (BMI) range of 19–40 Kg/m^2^ and clinically normal biochemical parameters. The inclusion criteria for IGT subjects was fasting glycaemia levels between 100 mg/dL and 125 mg/dL on at least two previous occasions and/or a HbA1c range of 5.7–6.4%. Type 2 diabetes was defined according to the American Diabetes Association guidelines [2]. Exclusion criteria were type 1 diabetes, malignant neoplasm, triacylglycerols (TG) > 400 mg/dL, macrovascular complications, patients with poorly controlled type 2 diabetes (HbA1c ≥ 8%) or who were being treated with insulin or intestinal disaccharidase inhibitors.

The study was conducted according to the guidelines laid down in the Declaration of Helsinki, and all procedures involving human subjects were approved by the university hospital Dr. Peset Ethics Committee on 28 March 2012 (Project code: FRUIT UP/01; Committee code: 11/12). Written informed consent was obtained from all patients.

This was a randomized, double-blind study, as previously described [17,21,22]. Each group of volunteers was then divided into two subgroups. One subgroup received a sucrose-enriched beverage (SEB) as a placebo and the other received a carob-pod, pinitol-enriched beverage (PEB; Fruit Up^®^; ADM&WILD-Valencia SAU, Valencia, Spain) [17,21,22]. The PEB consisted of a complex mixture of naturally-occurring soluble carbohydrates (including mono-, di-, oligo-saccharides and polyols, mainly pinitol, myoinositol plus D-chiro-inositol–soluble fibre) and minor compounds (organic acids, minerals, aminoacids) derived from carob pods. The sucrose-based beverage contained very similar amounts of non-polyol soluble carbohydrates, macronutrient composition and total number of calories to the PEB, but excluded inositols (Table 1). The sweetness and energy supply of inositols compared to other common sweeteners is showed in Supplementary Table S1. This trial was registered on clinicaltrials.gov under the study number NCT01754792. 

The anthropometric and biochemical profile of the study population has been previously reported [16,17,21,22] for healthy subjects, IGT subjects and in T2DM patients. (For further information, see Supplementary Material).

#### 2.1.2. Blood Collection and Sample Preparation

**Figure 1:**
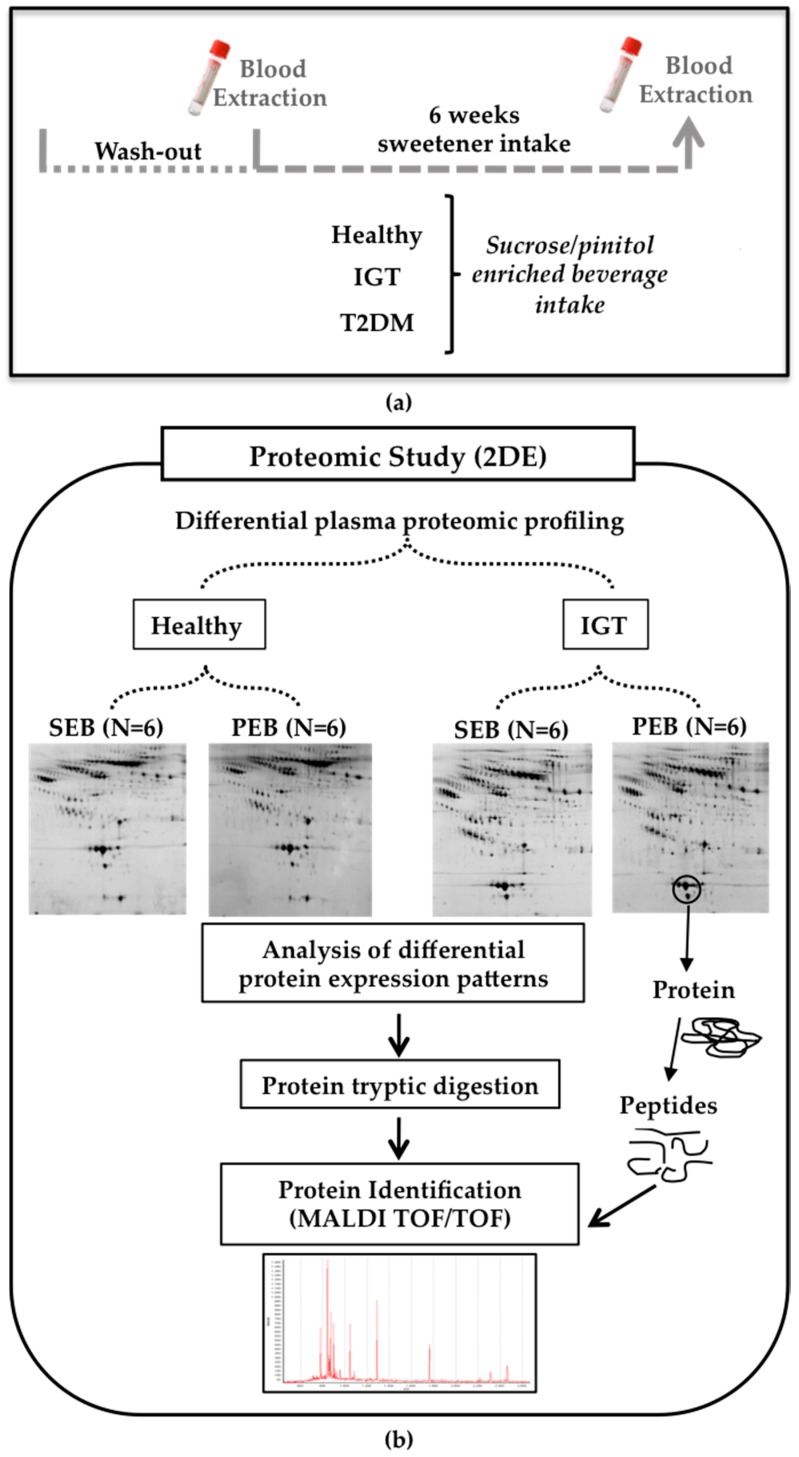
Human study design (**a**) Blood samples were obtained before and after six weeks of daily sucrose/pinitol-enriched beverage supplementation; (**b**) Study workflow. A proteomic approach was used to identify changes in the proteomic profile of the different plasma fractions of healthy and impaired glucose tolerance (IGT) volunteers after the intake of the sucrose/pinitol-enriched beverage (*N* = 6 each group).

Venous blood samples from the volunteers were collected by the volunteers at 8–10 am after 12 h overnight fasting at the moment of inclusion (T0) and after six weeks (T6) of intake of the supplemented beverages (Figure 1a; baseline and endpoint of the study). Blood samples were collected in anticoagulant-free Vacutainer tubes for serum preparation. Serum fractions were separated by centrifugation at 3.000× *g* at 4 °C for 20 min, aliquoted and then stored at −80 °C in Valencia [16,17,21,22]. Sample aliquots were transported in dry ice to Barcelona for the present mechanistic study. All samples were kept without thawing at −80 °C until used.

#### 2.1.3. Sample Preparation

For proteomic studies, serum samples were prepared as previously reported [23,24,25]. Briefly, samples were sonicated (six cycles of 15 s each) in ice and filtrated (0.22 µm) by centrifugation to avoid the presence of impurities. The six most abundant serum proteins were depleted using a specific affinity cartridge with binding capacity for albumin, IgGs, IgAs, transferrin, antitrypsin, and haptoglobin (Multiple Affinity Removal Spin Cartridge, Agilent Technologies, Santa Clara, CA, USA), as reported by the providers. Serum depleted samples (called the total serum fraction) were concentrated and desalted by centrifugation with 5 kDa cutoff filter devices and sample buffer was exchanged to a urea-containing buffer (8 M urea, 2% CHAPS). The protein concentration in the serum extracts was measured with a 2D-Quant Kit (GE Healthcare, Little Chalfont, UK). All processed samples were stored at −80 °C until used.

#### 2.1.4. Proteomic Analysis

The proteomic serum profile of six volunteers from each group and condition were randomly selected and analyzed by two dimensional gel electrophoresis (2-DE) and mass spectrometry (MS) (Figure 1b).

#### Two-Dimensional Gel Electrophoresis

A protein load of 100 μg (analytical gels) and 300 μg (preparative gels) of the urea/chaps serum extracts was applied to 17-cm dry strips (pH 4–7 linear range; BioRad). The second dimension was resolved in 10% SDS-PAGE (sodium dodecyl sulfate-polyacrylamide gel electrophoresis). Gels were developed by fluorescent staining (Flamingo; BioRad, Hercules, CA, USA). For each independent condition, 2-DE for protein extracts from the baseline and the post-intake of placebo or Fruit-Up^®^ were processed in parallel to guarantee maximum comparability. Analysis for differences in the protein profiles was performed with the PD-Quest 8.0 software (BioRad, Hercules, CA, USA). Each spot was assigned a relative value that corresponds to the single spot volume compared to the volume of all the spots in the gel, following background extraction and normalization between gels [26].

#### Mass Spectrometry Analysis

Proteins were identified after in-gel tryptic digestion and the extraction of peptides from the gel pieces, as previously described [24,27], by matrix-assisted laser desorption/ionization time-of-flight (MALDI-TOF) using an AutoFlex III Smartbeam MALDI-TOF/TOF (Bruker Daltonics, Billerica, MA, USA). Samples were applied to Prespotted AnchorChip plates (Bruker Daltonics, Billerica, MA, USA) surrounding the calibrants provided on the plates. Spectra were acquired with flexControl in reflector mode (mass range: 850–4000 *m*/*z*, reflector 1: 21.06 kV; reflector 2: 9.77 kV; ion source 1 voltage: 19 kV; ion source 2: 16.5 kV; detection gain: 2.37x), with an average of 3500 added shots at a frequency of 200 Hz. Each sample was processed with flexAnalysis (version 3.0, Bruker Daltonics, Billerica, MA, USA) considering a signal-to-noise ratio over three, applying statistical calibration and eliminating background peaks. For identification, peaks between 850 *m*/*z* and 1000 *m*/*z* were not considered. After processing, spectra were sent to the BioTools interface (version 3.2, Bruker Daltonics, Billerica, MA, USA) and a MASCOT server search on Swiss-Prot 57.15 database was carried out (taxonomy: homo sapiens, mass tolerance 50 to 100, up to two trypsin miss cleavages, global modification: carbamidomethyl (C), variable modification: oxidation (M)). Identified proteins were accepted when a mascot score higher than 52 was obtained by peptide mass fingerprint and confirmed by peptide fragmentation was working in the reflector mode.

#### Quantification of Protein Serum Levels

A commercial sandwich-based ELISA (enzyme-linked immunosorbent assay) kit was used to measure total IGF-I (Insulin-like growth factor-I) levels in the serum samples before and after the intake of PEB by the control (*N* = 20) and IGT volunteers (*N* = 20). In addition, a group of diabetic patients was included (*N* = 19). The detection range of the kit was 2.0–50 ng/mL (IGF-I ELISA E20, Human Insulin-Like Growth Factor-I ELISA Kit; Mediagnost, Budapest, Hungary).

### 2.2. Animal Study

The study protocol was approved by the institutional ethics committee “Animal Experimentation Committee of the Cardiovascular Research Centre (CSIC-ICCC)” conforming to the position of the American Heart Association (AHA) on Research Animal Use adopted by the AHA on 11 November 1984. All procedures fulfilled the criteria established by the “Guide for the Care and Use of Laboratory Animals” published by the United States National Institutes of Health (NIH; NIH Publication No.85-23, revised 1996).

Animals were housed in the ICCC (Instituto Catalán de Ciencias Cardiovasculares) animal facilities in a dedicated room with controlled temperature (22 ± 1 °C), humidity (60%), and lighting (12 h light–dark cycles).

#### 2.2.1. Animal Model

Male Zucker diabetic fatty (ZDF) rats (350 g ± 30 g; 12 weeks-old; *N* = 10) supplied by Charles River (Barcelona, Spain) were used as an animal model of severe T2DM. ZDF rats progress to T2DM due to insulin resistance and express abnormalities such as early hyperinsulinemia (that quickly decreases as the β-cells fatigue), hyperglycemia, glucose intolerance, hyperlipidemia, mild nephropathy and hyperleptinemia [28].

ZDF rats were fed a diet with high protein, carbohydrate and fat levels to develop diabetes (LabDiet^®^ 5008 Formulab, P.O. Box 19798 St. Louis, MO, USA). The study was conducted in rats under a pinitol-free diet to avoid potential interactions. Animals were provided with food and water ad libitum.

#### 2.2.2. Experimental Design

ZDF rats were randomized into two parallel groups (1:1) receiving the low-glycemic index pinitol solution (PEB; intervention group) or a sucrose solution (SEB; control group).

**Figure 2:**
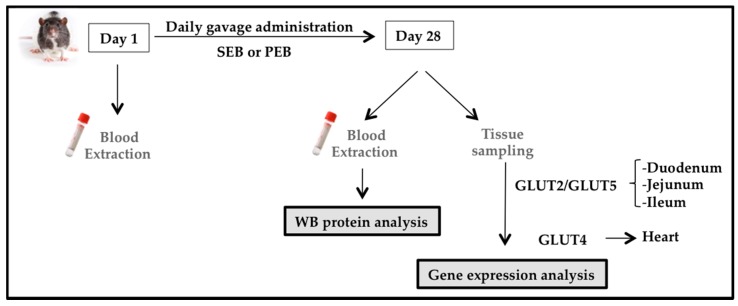
Zucker diabetic fatty (ZDF) rat study design. Blood samples were obtained at baseline and after 28 days of sucrose/pinitol-enriched beverage administration. Tissues were obtained, frozen in liquid nitrogen and stored at −80 °C until used.

Animals were administered a 1.15 g/kg carob-pod pinitol preparation, equivalent to a daily dose of pinitol in humans, as tested in previous studies [16]. The dose of sucrose was calculated in order to ensure that all animals received the same amount of non-polyol carbohydrates and total energy as those obtained by PEB intake (0.90 g/kg). Animals were administered daily for four weeks (28 days) by gastric gavage of either sucrose or pinitol solution. On days 1 and 28 (after a 4-h time period of food deprivation) blood was collected from the tail vein, for the blood glucose level analysis (Glucocard™ Memory 2 meter, Menarini Diagnostics, Firenze, Italy; Figure 2). Animals were kept conscious in order to avoid potential transient hyperglycemia caused by sedation [29].

Upon completion of the experiment on day 28, the animals were deeply anesthetized with a mixture of ketamine (75 mg/kg) + medetomidine (0.5 mg/kg) + buprenorphine (0.1 mg/kg) and euthanized with an intracardiac injection of a pentobarbital overdose (400 mg/kg). A portion of duodenum, jejunum, ileum, large intestine and the heart were extracted and cleaned to avoid bacterial RNAse interference, immediately frozen in liquid nitrogen and stored at −80 °C until processed.

#### 2.2.3. Western Blot Analysis

Protein extracts were resolved by 1-DE under reducing conditions and electrotransferred to nitrocellulose membranes in semi-dry conditions (Trans-Blot Turbo system; BioRad, Hercules, CA, USA). C4A complement detection was performed using a rabbit policlonal antibody against a recombinant fragment corresponding to a region within amino acids 23-302 of the protein (NBP2-14,893, 1:1000 dilution, Novus bio, Littleton, CO, USA). Band detection was performed using a chemiluminiscent substrate dye (SuperSignal^®^ West Dura Extended Duration Substrate, Thermo Scientific, Waltham, MA, USA) and a molecular imager ChemiDoc XRS System, Universal Hood II (BioRad, Hercules, CA, USA). Band quantification was performed with Image Lab 4.0 software (BioRad laboratories, Hercules, CA, USA). The protein load was normalized with total protein staining, as previously described [27].

#### 2.2.4. Glucose Transporter mRNA Expression

We evaluated the potential effect of the intake of PEB on the expression of three main glucose transporters: GLUT2, GLUT4 and GLUT5. Due to the tissue specificity and pinitol absorption, we measured GLUT2 and GLUT5 in the duodenum, jejunum and ileum, and GLUT4 in the heart. To this end, the aforementioned samples were pulverized in liquid nitrogen and mRNA was isolated using the Tripure^TM^ Isolation Reagent (Roche Molecular Biochemicals, Pleasanton, CA, USA) according to the manufacturer’s instructions and then gene expression was assessed by real-time PCR analysis; GLUT2 (Rn00563565, Thermo Fisher Scientific, Waltham, MA, USA), GLUT4 (Rn01752377, Thermo Fisher Scientific, Waltham, MA, USA) and GLUT5 (Rn00582000, Thermo Fisher Scientific, Waltham, MA, USA).

### 2.3. Statistical Analysis

Data are expressed as mean and standard error (SEM) unless stated. *N* indicates the number of subjects tested. Statistical analysis was performed with Stat View 5.0.1 software (SAS Institute, Cary, NC, USA). Differences between the basal condition and after six weeks of intake of the pinitol-enriched beverage were tested using a repeated measurement Willcoxon analysis. Differences between groups were tested with a nonparametric Mann–Whitney test. A *p*-value ≤ 0.05 was considered significant.

## 3. Results

### 3.1. Pinitol-Enriched Beverage Intake Induces Serum Proteomic Changes in Proteins Related to the Insulin Secretion Pathway

Six weeks of pinitol-enriched beverage intake induced a significant decrease in glucose levels both in IGT and healthy subjects (*P* = 0.03). On the contrary, sucrose intake induced an increase in glucose levels in both groups of volunteers (*P* = 0.03; Table 2; Supplemental Figure S1).

In order to investigate the proteins involved in the observed glucose-lowering-effect of PEB, we analyzed the serum proteomic profile of both IGT and healthy subjects at baseline and after six weeks of pinitol or sucrose-enriched beverage intake. 

**Figure 3:**
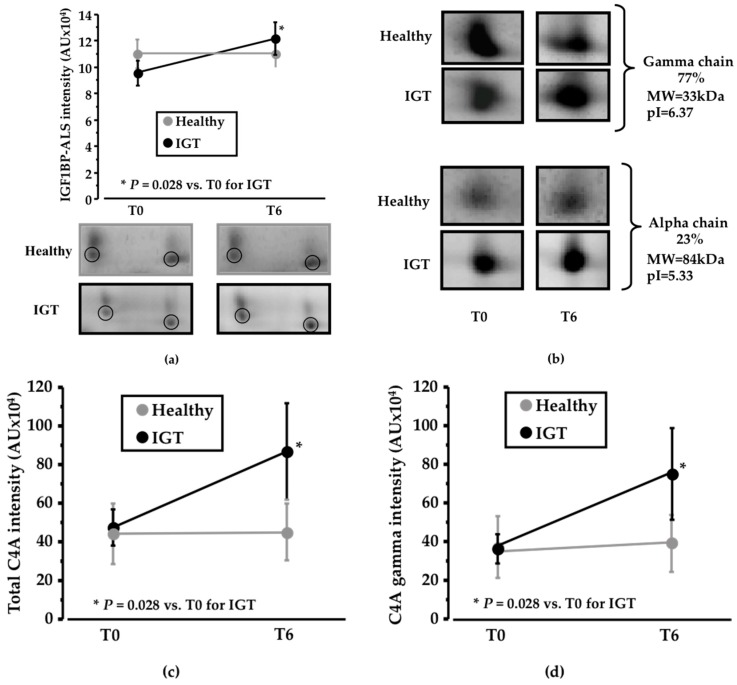
Changes in the proteomic profile. (**a**) Line diagram and representative 2-DE (two-dimension electrophoresis) images showing IGF1BP-ALS (Insulin-like growth factor-1 binding protein acid labile subunit) change after six weeks of sucrose/pinitol-enriched beverage intake. Change in IGF1BP-ALS level was higher in IGT patients compared to healthy subjects (*N* = 6; *P* = 0.200) is observed in IGT patients; (**b**) Representative 2-DE images of C4A complement alpha and gamma chain. Alpha chain corresponds to the 27% of the total C4A complement identified complement. Gamma chain represents the 77% of the total C4A complement identified protein (**c**) Line diagram showing total C4A complement change of both healthy and IGT volunteers after the intake of the pinitol-enriched beverage. A significant change increase of the C4A complement level (*N* = 6; *P* = 0.02) is observed on IGT patients; (**d**) Line diagram showing C4A complement gamma chain of both healthy and IGT volunteers after the intake of the pinitol-enriched beverage. A significant change increase of C4A complement level (*N* = 6; *P* = 0.02) is observed in IGT patients.

Among the identified proteins, IGF1BP-ALS (IGF1—insulin-like growth factor 1; BP—binding protein; ALS—acid labile subunit; MW = 63kDa; pI = 6.13) was detected in the 2-DE analysis as a cluster of two different spots that showed an increased change in IGT volunteers after PEB intake compared to sucrose intake (*P* = 0.028; Figure 3a). On the contrary, no differences were detected in the healthy subjects (Supplemental Table S1).

Together with changes in IGF1BP-ALS, complement C4A also showed a significant shift in its distribution profile in IGT subjects, but not in healthy subjects. C4A was identified as two different chains, the gamma chain (MW = 33 kDa; pI = 6.37), which corresponds to 77% of total protein intensity, and the alpha chain (MW = 84kDa; pI = 5.33), that represents 23% of total protein intensity (Figure 3b). Total C4A levels showed a significant increase (*P* = 0.028; Figure 3c) in IGT subjects after six weeks of dietary supplementation with PEB compared to sucrose intake. No differences were found in the alpha chain, whereas a significant increase in the IGT subjects after the intake of the PEB was observed in the gamma chain (*P* = 0.028; Figure 3d) when compared to sucrose intake. On the contrary, no differences in C4A expression were detected in the healthy group (Supplemental Table S1).

### 3.2. Potential Mechanisms Involved in the Peb Glucose Modulating Effect

**Figure 4:**
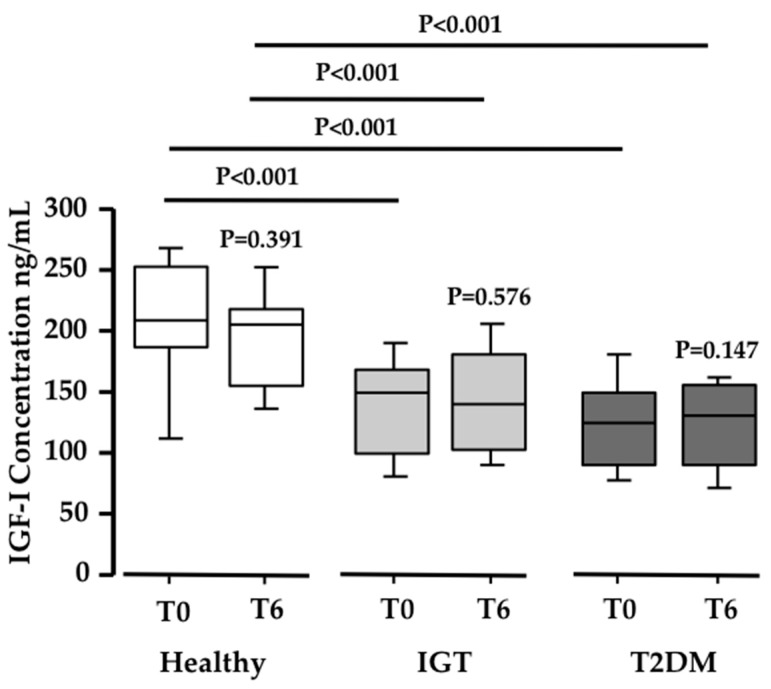
Changes in IGF-1 levels. Box-plot diagram showing the significant decrease in IGF1 concentration (ng/mL) in IGT and T2DM volunteers when compared to healthy subjects (*N* = 20; 20; 19 respectively; *P* ≤ 0.0005).

Since the acid labile subunit (ALS) was found increased in the proteomic analysis, and ALS binds IGF-1 [30], we measured the total IGF-1 serum levels in the three study populations. Interestingly, both the IGT and T2DM subjects showed significantly lower basal serum levels of IGF-1 when compared to healthy subjects (*N* ≥ 19 in each group; *P* < 0.001; Figure 4). However, no differences were observed in IGF-1 serum levels after PEB intake in any of the three groups. This means that PEB intake, while regulating ALS, has no influence on IGF-1 levels.

To better understand the mechanisms related to the observed glucose reduction produced after the intake of a pinitol-enriched beverage, ZDF rats, as an animal model of diabetes, were included in the study.

**Figure 5:**
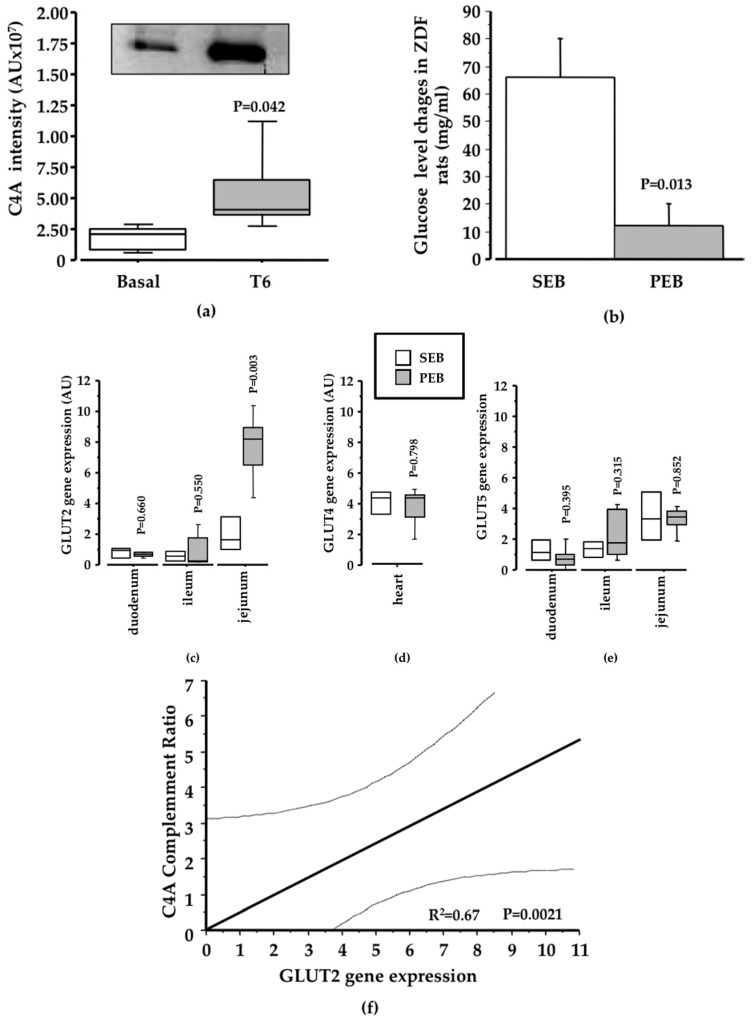
Results validation. (**a**) C4A profile in ZDF rat samples. Box-plot and representative western blot image showing changes in C4A intensity in ZDF rats supplemented with a pinitol-enriched beverage (*N* = 5; 1.21-fold change; *P* = 0.04); (**b**) In ZDF rats, the blood glucose level change after SEB intake was significantly higher than blood glucose level changes after four weeks of PEB intake (*N* = 5; *P* = 0.01); (**c**) Changes in GLUT2 levels. Box-plot diagram showing the change of the gene in the small intestine. A significant reduction in GLUT2 gene expression in the jejunum of ZDF diabetic rats supplemented with pinitol was achieved when compared with the intake of a sucrose-enriched beverage (*N* = 9; *P* = 0.003); (**d**) No change was observed in GLUT4 after pinitol-enriched beverage intake; (**e**) No change in GLUT5 was achieved after pinitol-enriched beverage intake; (**f**) A significant positive correlation was observed between the increase of C4A intensity and the level of GLUT2 achieved (*P* = 0.002).

Changes in C4A serum levels were confirmed in ZDF rats by Western blot analysis, showing a significant increase in this protein after four weeks of pinitol intake compared to basal levels (*P* = 0.042; Figure 5a). Leptin-receptor-deficient ZDF rats acquire a very severe phenotype, reaching an average of 400 mg/dL glucose. Interestingly, sucrose feeding induced an increase in 100 mg/dL, while the pinitol solution did not impair glycaemia (Figure 5b). To further explore the potential mechanisms behind the observed Fruit-Up^®^-mediated effects, we analyzed the gene expression levels of three different glucose transporters (GLUT2, GLUT4 and GLUT5) at their major sites of expression. A significant increase of GLUT2 after dietary supplementation with the pinitol solution when compared to sucrose intake was observed in the jejunum (*P* = 0.003; Figure 5c). However, no change was observed either in GLUT4 (Figure 5d), or in GLUT5 (Figure 5e). The observed change in C4A protein levels showed a direct correlation with GLUT2 expression levels in the jejunum (*P* = 0.002; Figure 5f).

## 4. Discussion

The refined sugar intake steadily increased in the last years and it is nowadays the most commonly used sweetener for foods and beverages contributing to the high glycaemic indices of this diet [31]. High glycaemic index diets have been linked to the development of diverse metabolic diseases such as obesity, decreased insulin sensitivity, an elevated risk of developing diabetes [32] and are a risk factor for coronary heart disease [33]. Thus, the reduction of glucose levels is one of the main objectives to prevent and treat diabetes [34].

Previous studies with pinitol-enriched beverages have demonstrated a significant reduction in the post-prandial glycaemic response and glucose levels both after 24 h [16,35] and after 12 weeks of daily consumption [17] in healthy volunteers, when compared with a sucrose-based beverage. Also, its daily oral ingestion positively impacts insulin resistance [22].

Here, we have analyzed the serum proteomic profile in IGT and in healthy subjects after the intake of the pinitol-enriched beverage (PEB), to investigate the mechanisms associated with the improvement in the glycaemic response previously observed. With this aim, pair comparisons of protein-intensity values between basal conditions (before intervention) and after six weeks of PEB/SEB intake were performed for each individual. This proteomic study was based on 24 subjects randomly chosen among the total study population (six/study group) and the suitability of the groups was based on the consistent response of the plasma glucose levels to the interventions. Importantly, a significant change in the two proteins closely related with β-cell insulin secretion, IGF1BP-ALS and C4A, was observed only in IGT volunteers. IGT patients have an altered glucose metabolism compared to healthy subjects, supporting the view that IGT patients have different responses to glucose metabolism than healthy subjects. However, the findings of this proteomic approach should be further assessed in new prospective studies specifically designed to this aim. 

**Figure 6:**
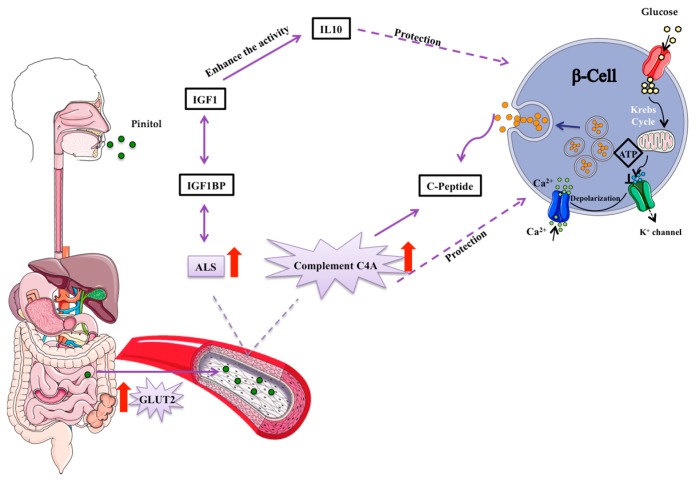
Suggested mechanism of pinitol-induced effects on the insulin secretion pathway. Pinitol is a glucose mimetic molecule, which is absorbed in the small intestine and distributed though the bloodstream to the whole organism. An increase in the acid labile subunit (ALS) and C4A complement proteins induce a protection of β-cells. Pro-preinsulin granules are packed inside pancreatic β-cells and secreted outside the cell as insulin, in response to nutrient intake.

There are multiple β-cell-related factors involved in impaired insulin secretion, such as the reduction of β-cell mass, glucotoxicity and lipotoxicity [36]. Insulin is packed into β-cells in small granules like propreinsulin, the first inactive form of insulin, with an hexameric crystalline structure, formed by six insulin units [37]. Most diabetic patients have already lost 70–80% of their original β-cell mass [38]. Each β-cell contains more than 10,000 granules, so its protection is an important strategy to prevent impaired insulin secretion [39,40]. These granules are then secreted from β-cells as proinsulin, which is composed of an insulin chain attached to C-peptide (Figure 6). IGF-1 reduces β-cell inflammation, protecting from apoptosis and promoting proliferation [41]. Therefore, the relationship of IGF-1 with the prognosis of T2DM has been widely studied. In fact, low levels of IGF-1 are related with obesity and impaired glucose tolerance [42]. In addition, IGF-1 is related with C-peptide secretion and β-cell regeneration (Figure 6) and low IGF-1 levels are associated with reduced insulin secretion and can be an important marker of β-cell function [43]. In this context, the search for strategies to maintain the levels of IGF-1 is very important to preserve β-cell function. In line with this, we found decreased IGF-1 serum levels in IGT and T2DM subjects. Importantly, IGF-1 is mainly found in a complex with one of its binding proteins (BP) and with the acid-labile subunit (ALS) [44]. ALS is an essential component to maintain the integrity of IGF-1 [44]. IGF-1 as a free molecule has a half-life shorter than 12 min, whereas this rises to 12 h when forming a complex with ALS and its BPs [30,45,46]. In this respect, ALS deficiency in humans has been characterized by a severe reduction in IGF-1 levels and insulin insensitivity [44]. Here, we have observed that the regular consumption of the pinitol-enriched beverage induced a significant increase in ALS levels in those volunteers suffering impaired glucose resistance. These results suggest that the intake of a pinitol-enriched beverage might help maintain IGF-1 levels by increasing levels of ALS, therefore improving IGF-1 stability in patients with impaired glucose metabolism. Indeed, nowadays it is recognized that the importance of a protein in a pathology lies not only in the total levels, but also in its stability, conformation and ability to make up complexes.

Besides changes in IGF1BP-ALS, an increase in C4A complement was also observed. C4A is a non-enzymatic component of the complement system, which takes part in the initial steps of the complementary pathway. The complement system is involved in several processes including the dysregulation of adipose tissue metabolism, inflammation, endothelial dysfunction and insulin resistance [47,48]. The relationship between C4A and the progression and prognosis of T2DM has not been widely studied. However, the relationship between C4A levels and insulin-dependent DM has been previously demonstrated [49,50]. In fact, low levels of this protein appear after high glucose intake [51]. Also, higher levels of C4A are associated with higher levels of C-peptide (Figure 6), indicative of enhanced insulin secretion [52,53]. C4A protein has been also suggested to protect β-cells [53] and it is involved in innate immunity [50,54,55]. With all this in mind, we sought to investigate the potential mechanism behind the increase in C4A protein levels induced by the intake of a pinitol-enriched beverage. For this purpose, we used leptin-receptor-deficient ZDF rats as an animal model of highly severe T2DM and compared those receiving PEB with those treated with sucrose solution, used as control group. While in those rats receiving sucrose a significant increase in blood glucose levels was observed, in those rats that received the PEB, glucose levels remained at the baseline. Higher levels of C4A were also found in ZDF rats after the intake of a pinitol solution when compared to sucrose intake, confirming the results of the proteomic study in humans and suggesting a potential implication of this protein in the regulation of blood glucose levels induced by the administration of the pinitol solution. β-cell protection achieved by C4A has been related to glucose transport across the plasma membrane [53]. Interestingly, we observed a significant increase in the expression of the glucose transport 2 (GLUT2) in jejunum after the regular intake of pinitol. This glucose transport is located in multiple organs and tissues, the small intestine, the pancreas, the liver and the brain. GLUT2 plays a very important role in glucose homeostasis control by an inter-organ communication system [56], including the passive movement of glucose through the cellular membrane of β-cells [57,58]. Importantly, we found that the increase in GLUT2 expression in the jejunum of ZDF rats, where pinitol is absorbed to be further distributed through the whole organism, was directly associated with the increase in C4A protein levels after pinitol intake, which suggests a protective effect on the pancreatic beta cells [53], explaining the significant difference in glucose levels after PEB and SEB interventions. In severely advanced T2DM states, as occurs in our animal model, multiple alterations within several metabolic signalling pathways, including the desensitization of GLUT2 translocation and the disruption of the insulin regulation of glucose absorption may developed. The enzyme glucokinase and GLUT2 comprise the glucose sensing system, regulating insulin secretion from pancreatic β-cells in response to nutrient intake and, therefore, preventing excessive blood glucose transport after a sugar-rich meal [59,60]. Of note, regular intake of the pinitol-enriched beverage was demonstrated to exert an insulin-mimetic activity in healthy subjects without affecting insulin secretion [16]. In fact, previous recent studies have shown the favourable effects of pinitol on the treatment of insulin-related disorders because of its insulin-like function (e.g., reduces blood glucose levels in T2DM patients) [35,61]. In this regard, we observe that the regular intake of pinitol induces a coordinated increase in C4A and GLUT2 in ZDF rats, helping to improve their metabolic system.

Diabetes mellitus is associated with β-cell destruction, so its regeneration and protection remain key strategies for the prognosis of the disease [62]. Our results suggest for the first time that the substitution of a normal sugar source with a naturally-based, pinitol-enriched beverage in IGT subjects could help to attenuate glucose metabolism disruption by inducing a coordinated change in C4A complement and IGF1BP-ALS, two important molecules with direct implications on β-cell survival. Furthermore, changes in C4A complement seem to be related to an increase in the expression of the glucose transporter GLUT2 (Figure 6). Future studies on the differential pattern of glucose transporters in various tissues, including liver and pancreas, would help the better understanding of the mechanisms directly associated with the beneficial effects on glucose metabolism after PEB intake. Therefore, we propose that the substitution of sucrose by carob pod sweetener may help efforts to normalize an impaired glucose metabolism, and to reduce the risk associated with the intake of processed sugars.

### Limitations of the Study

The manuscript referred to a proteomic discovery design aimed at identifying the differential protein signatures associated with the glycaemic pattern after the regular and chronic ingestion of a pinitol-enriched beverage. Specifically, the main goal of the study was to highlight proteins potentially involved in the glycaemic response to the intervention with pinitol. However, further studies are needed to better explain mechanisms and pathways associating the pinitol intervention with changes in glucose metabolism. Thus, measuring GLUT2 in pancreas tissue would provide extra information on the beta-cell response to pinitol intake. Unfortunately, we do not have pancreas tissue to perform this analysis.

## Figures and Tables

**Table 1:** Pinitol/sucrose-enriched beverage characterization.

	PEB	SEB
Pinitol (g)	4.00	-
Myoinositol + D-chiro-inositol (g)	0.45	-
Sugars(g)	34.90	42.5
-Glucose	6.23	-
-Fructose	4.83	-
-Sucrose	37.29	42.5
-Others	0.58	-
Oligosaccharides (g)	0.05	-
Soluble fibre (g)	1.65	-
Total carbohydrates (g)	41.18	42.50
Total available carbohydrates (g)	39.12	42.50
Total calories (kcal)	155.0	170.0

Nutritional composition of the pinitol/sucrose-enriched beverage per 500 mL (daily dose).

**Table 2:** Biochemical characteristics of the subjects selected for the proteomic study.

	Healthy Subjects						IGT Subjects					
	SEB (*N* = 6)			PEB (*N* = 6)			SEB (*N* = 6)			PEB (*N* = 6)		
	T0	T6	*P* Value	T0	T6	*P* Value	T0	T6	*P* Value	T0	T6	*P* Value
Cholesterol (mg/dL)	192	202	0.022 *	183	178	0.398	197	203	0.602	189	194	0.798
cLDL(mg/dL)	112	125	0.004 *	109	108	0.706	104	130	0.266	122	125	0.824
cHDL (mg/dL)	54	52	0.283	55	52	0.047 *	54	53	0.734	42	43	0.812
TG (mg/dL)	126	125	0.934	88	89	0.802	111	101	0.379	121	128	0.797
Urea (mg/dL)	24	26	0.168	32	34	0.445	41	35	0.028 *	43	35	0.01 *
Glucose (mg/dL)	87	92	0.057	98	89	0.003 *	100	103	0.003 *	120	105	<0.001*

T0 corresponds to the day of inclusion in the study and T6 six weeks after the intake of pinitol-enriched beverage (PEB) or sucrose-enriched beverage (SEB). * Significant change T0 vs. T6. *P* value ≤ 0.05.

## Supplementary Materials

The following are available online at http://www.mdpi.com/2072-6643/10/3/271/s1, Figure S1: Cell line chart showing the individual glucose change of each subject after 6 weeks of PEB or SEB supplementation, Table S1: Sugars characterization, Table S2: Changes in the proteomic profile after SEB intake.
