# Supplementary material for: Effects of a Carob-Pod-Derived Sweetener on Glucose Metabolism
Source: Nutrients. 2018 Feb 27;10(3):271. doi: 10.3390/nu10030271 (PMC5872689; doi:10.3390/nu10030271)
Supplement: Supplementary file 1 [file nutrients-10-00271-s001.pdf]

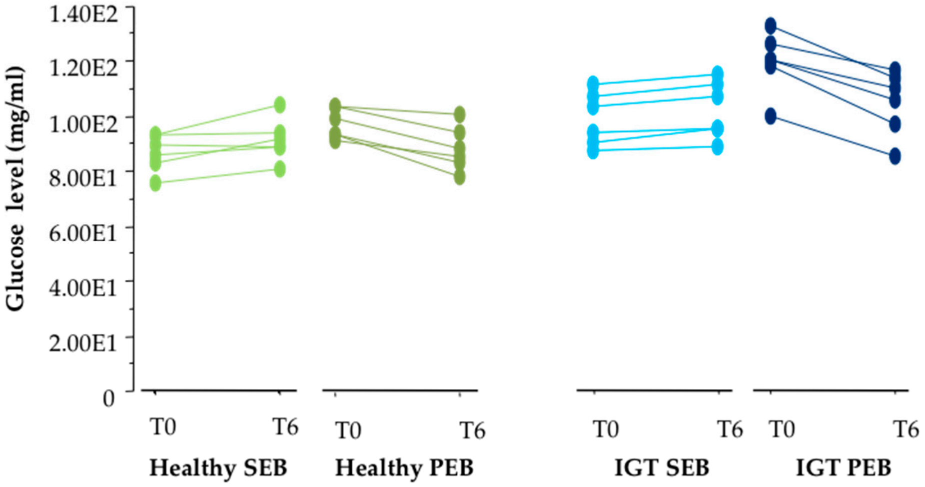

Figure S1. Cell line chart showing the individual glucose change of each subject after 6 weeks of PEB or SEB supplementation.

Table S1. Sugars characterization [1,2].

Product	Relative Sweetness *	Kcalories/g
Pinitol	50%	2.5 Kcal/g
Sucrose	100%	3.9 Kcal/g
Fructose	150%	3.7 Kcal/g
Glucose	40%	3.8 Kcal/g

* Taking sucrose as 100% of sweetness.

Table S2. Changes in the proteomic profile after SEB intake.

	Healthy Subjects			IGT Subjects		
	**T0**	**T6**	**Δ%**	**T0**	**T6**	**Δ%**
**C4A alpha**	6.2 ± 0.8	8.9 ± 1.6	44.6	12.5 ± 1.6	12.0 ± 2.0	-4.0
**C4A gamma**	47.2 ± 12.4	56.2 ± 10.2	19.2	59.6 ± 7.0	48.3 ± 16.3	-19.1
**C4A Total**	53.4 ± 12.3	65.2 ± 10.2	22.1	72.2 ± 7.5	60.3 ± 16.3	-16.4
**IGFBP1-ALS**	31.3 ± 3.7	55.9 ± 25.2	78.5	36.9 ± 2.4	40.4 ± 1.9	9.4

*Data expressed as mean value x10^4 ± SEM. No significance was observed either in the healthy group or in the IGT group after the intake of a sucrose-enriched beverage.

[1] Dinicola, S.; Minini, M.; Unfer, V.; Verna, R.; Cucina, A.; Bizzarri, M. Nutritional and acquired deficiencies in inositol bioavailability. Correlations with metabolic disorders. *Int. J. Mol. Sci.* 2017, *18*.
[2] Aeberli, I.; Hochuli, M.; Gerber, P. A.; Sze, L.; Murer, S. B.; Tappy, L.; Spinas, G. A.; Berneis, K. Moderate amounts of fructose consumption impair insulin sensitivity in healthy young men: A randomized controlled trial. *Diabetes Care* **2013**, *36*, 150–156, DOI:10.2337/dc12-0540.
